# Impairing of Serotonin Synthesis by P-Chlorphenylanine Prevents the Forgetting of Contextual Memory After Reminder and the Protein Synthesis Inhibition

**DOI:** 10.3389/fphar.2018.00607

**Published:** 2018-06-12

**Authors:** Irina B. Deryabina, Lyudmila N. Muranova, Vyatcheslav V. Andrianov, Khalil L. Gainutdinov

**Affiliations:** Laboratory of Neuroreabilitation of Motor Disorders, Institute of Fundamental Medicine and Biology, Kazan Federal University, Kazan, Russia

**Keywords:** serotonin (5-HT), p-CPA, consolidation, contextual memory, reconsolidation, anisomycin (AN), snail

## Abstract

**HIGHLIGHTS**

- The injection of p-chlorophenylalanine, specific blocker of 5-HT synthesis 3 days before reminder with anisomycin administration prevented forgetting.

It is known that the reminder cause reactivation of the long-term memory and it leads to reconsolidation of memory. We showed earlier that the disruption of the reconsolidation of contextual memory in terrestrial snail was caused by anisomycin, the inhibitor of protein syntheses (Gainutdinova et al., 2005; Balaban et al., 2014). In this paper we investigated the possible changes of the memory reconsolidation under the conditions of serotonin deficit, caused by administration of p-chlorophenylalanine, the inhibitor of tryptophan hydroxylase synthesis (intermediate stage of the synthesis of serotonin). It was shown that the forgetting process for contextual memory after reminder and inhibition of protein synthesis did not occur if the serotonin transmission in nervous system was impaired. This effect was significantly different from the direct action of anisomycin, which blocked the reconsolidation of contextual memory. We concluded that the serotonin system was included to the process of memory reconsolidation.

## Introduction

Until recently it was believed that long-term memory about passed events is a trace, which is unchangeably stored in the brain as the memory in the computer's box. In recall, the brain addresses to this box and retrieves the data, but one memory triggers another and so formed the complex sequence with which we can better predict and understand the events taking place around us. During the conversion of memory from short-form to long-term phase, it is unstable immediately after receiving new information, but it becomes stable over time. This phenomenon is called memory consolidation. It has been shown that this stage requires gene expression and new protein synthesis (McGaugh, 2000; Sara and Hars, 2006; Balaban and Korshunova, 2011). The consolidated long-term memory can undergo to reorganization through different times after training. This result is achieved not only by reminder (the presentation to trained animal one of the components of training situation), but also by remembering, depending on the animal's state (Sara, 2000). Memory reactivation does not occur without “reminders” (Sara, 2000; Anokhin et al., 2002; Nader, 2003; Dudai, 2004; Alberini, 2005).

Recollection of memory is not simply the reproduction but reconstruction, trying to retrieve the passed event. That is, in each time the memories we not only express information, but, perhaps, create it again, this means that memory is a dynamic process and it either amplifies or alters during recalling (Schneider and Sherman, 1968; Lewis, 1979; Przybyslawski and Sara, 1997; Anokhin et al., 2002; Pedreira et al., 2002; Duvarci and Nader, 2004; Sara and Hars, 2006; Balaban, 2017). This process of repeating consolidation of memory after reminding called reconsolidation, which also requires protein synthesis (Sara, 2000; Mamiya et al., 2009; Nader and Hardt, 2009; Soeter and Kindt, 2013; Balaban et al., 2014). Temporal dynamics of memory reconsolidation depends from several parameters, including the age of the memory, so that weaker memories are easier reconsolidated, than the stronger memories (Suzuki et al., 2004; Mamiya et al., 2009; Alberini, 2011; Soeter and Kindt, 2013). The literature results demonstrate that the reactivation of long-term memory on the freezing of the rats returns it to a labile state, during which the injection of anisomycin shortly after reactivation produces amnesia on later tests, for a period from 1 to 14 days after reactivation (Nader et al., 2000; Duvarci and Nader, 2004; Nader and Hardt, 2009). Specific contextual learning and memory about it were also found in invertebrates (Child et al., 2003; Gainutdinova et al., 2004, 2005; Kemenes et al., 2006; Lukowiak et al., 2007; Solntseva et al., 2007; Cai et al., 2012; Balaban et al., 2014; Nikitin et al., 2016).

It is established that serotonin (5-HT) is a basic neurotransmitter for defensive behavior in mollusks and learning on the basis of defensive reflexes (Balaban et al., 1987; Glanzman et al., 1989; Gainutdinov et al., 1999; Il-Han et al., 2010; Bogodvid et al., 2017). In behavioral experiments it was shown that the disruption of serotoninergic system by the neurotoxin 5.7-DHT did not change the original memory, however, led to a memory impairment after repeated reactivation (Balaban et al., 2016). An unavailability of reactivation under the action of the antagonist of serotonin receptors methiothepin was also shown (Nikitin and Solntseva, 2012). These results show relevance of analysis of long-term memory after inhibition of 5-HT synthesis. One of the drugs that causes depletion of brain 5-HT is p-chlorophenylalanine (p-CPA) (Reader and Gauthier, 1984).

Therefore, based on the literature data, we set the task to study the dependence of the reconsolidation of contextual memory in the snail on serotonin, using the p-CPA tryptophan hydroxylase blocker to disrupt the synthesis of 5-HT.

## Materials and methods

### Experimental animals

The terrestrial snails *Helix lucorum* from the Crimean population, were used in the experiments. Snails most time were stored asleep (nonactive state). Prior to the experiments, the snails were kept for no less than 2 weeks in a glass terrarium in a humid atmosphere at room temperature in the active state (they were crawling, ate food). All groups were housed in separate terrariums which were kept together all the time in the same room under the same conditions. The animals with approximately the same weight (about 25 g) were selected. Two days before the training session the experimental animals were deprived of food. This series of experiments was carried out at different seasons (from January to February, from September to October). The results obtained in both seasons were similar. The 55 terrestrial snails *Helix lucorum* were used in the experiments.

### Drugs and injections

The effective blockade of protein synthesis by AN was demonstrated in identified neurons of terrestrial snail Helix (Ghirardi et al., 2004). Therefore solutions of AN (anisomycin (2-[p-Methoxybenzyl]-3,4-pyrrolidinediol 3-acetate, Sigma) were used in this study for protein blockade.

P-chlorophenylalanine is one of the various drugs which depress tryptophan hydroxylase. It is the first and presumably rate-limiting enzyme in 5-HT biosynthesis (Bloom and Giarman, 1968; Park et al., 1994). P-CPA caused the depletion of brain 5-HT (Koe and Weissman, 1966; Reader and Gauthier, 1984; O'Leary et al., 2007). It was shown that p-CPA after an intraperitoneal injection in doses 100, 200, and 300 mg/kg caused a dose-dependent decrease in cortical content of 5-HT in 3 times after 24 h and in 6–9 times through 2 and 4 days (Pappius et al., 1988). The decrease of 5-HT in the brain of rats after intraperitoneal treatment of p-CPA gradually occurs, reaching its maximum by the third day, and remains low at least during the week (Popova et al., 1978). The optimal dose from these studies should be 200 mg/kg. We found earlier that p-CPA in these doses caused a disruption of defensive reflex conditioning in terrestrial snail (Gainutdinov et al., 1999). Therefore the p-CPA (DL-4 - Chlorophenylalanine ethyl ester hydrochloride 97%, Sigma) was used for the inhibition of 5-HT synthesis.

Intracoelomic (intragemocel) injections were performed with a fine needle via a non-sensitive part of the foot skin normally (the region of the sinus node) hidden under the shell (Gainutdinova et al., 2005; Balaban et al., 2014). During injections, the snails stopped locomotion and lowered the ommatophores, mostly because the shell was fixed by the experimentator, but never showed a generalized withdrawal into the shell. The solutions of AN were injected at a dose of 0.4 mg/snail (dissolved in 0.2 ml of saline for snail–SS). The solutions of p-CPA were injected at a dose of 0.2 mg/kg (dissolved in 0.1 ml of SS).

### Contextual learning

The conditioned situation reflex in contextual paradigm “on the ball” was developed in a situation when the animals were rigidly fixed through their shells. In so doing it was preserved the freedom of movement of snails over the surface of a ball floating in the water and the snails was completely elongated out of the shell. The training consisted in the presentation of the unconditioned stimulus (electrical stimulation) when the snails were placed in a different context, such as on the ball. The 5 electrical stimulations per day (1–2 mA, 1 s, 50 Hz) were presented to snails within 5 days at their location on a ball for contextual learning by touching of two macroelectrodes: dorsally to the front of the foot and to the tail (Gainutdinova et al., 2005). The time from placing the animal in training context before the first stimulus and also between subsequent stimuli was approximately 15–20 min. The intensity of stimulation current was chosen large enough to start a defensive reaction related to the retraction of the front part of the foot and was about 2 mA. Used current did not cause any damage of animal's skin, which may appear as pigmented areas under an application of larger current (Gainutdinov and Beregovoi, 1994). The procedure of elaboration of conditioned context reflex lasted 5 days, during this time the snails have not received food. Food deprivation of invertebrates during the elaboration of conditioned situation reflexes is a standard technique, it is not related to the metabolisms of certain substances, and is determined by necessity of the active state of the animal (Nikitin and Solntseva, 2012; Balaban et al., 2014, 2016; Nikitin et al., 2016).

### Testing

Before the start of elaboration of the conditioned situation reflex, after learning and in processes of following treatments the testing of the level of defensive reaction as an indicator of formed long-term memory was performed. To do this, the amplitude of retraction of ommatophores in response to tactile stimulation of the anterior part of foot in percentage was measured. The maximum retraction of ommatophores was taken as 100% and it was recorded how much the snail withdraw ommatophore (0, 10, 25, 50, 75, 90, or 100%).

Testing was carried out in two contexts: during the free crawling of animals on a flat surface (glass cover of the aquarium) and in the situation of learning context (on the ball) (Gainutdinova et al., 2004). Each test consisted of 5 presentations of tactile stimulus (the time between the tests 7–10 min). The testing stimuli were first presented on the surface, and then the animals were moved to the ball and were tested there. The results were averaged in each context, and the average value of the level of defensive reaction in different context was compared. The time between the last test on the flat surface and the first test on the ball was 15–20 min. The tests were conducted visually and recorded on video.

**Figure 1:**
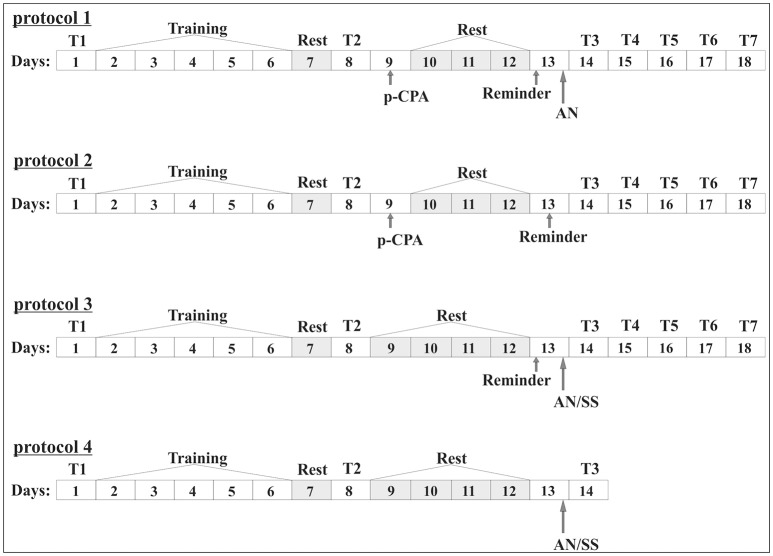
Scheme of the experiment (numbers 1, 2, 3). In the boxes indicated the days of the experiment. **General part:** 1 day (**T1**), initial testing of the level of defensive reactions; 2–6 days; (**Training**), development of conditioned reflex in context paradigm; Day 7 (**Rest**), rest; Day 8; (**T2**), testing of the level of defensive reaction after the procedure of training and rest; (**training**): **Protocol 1**. Group 1. “p-CPA + reminder + AN”: Day 9, injection of **p-CPA**; 10–12 days (**Rest**), rest; 13 day (**Reminder**), reminder session and subsequent injection of anisomycin (AN); 14–18 days (**T3, T4, T5, T6, T7**), testing of the level of defensive reaction after reminder session: **Protocol 2**. Group 2. “p-CPA + reminder”: Day 9, injection of **p-CPA**; 10–12 days (**Rest**), rest; 13 day (**Reminder**), reminder session; 14–18 days (**T3, T4, T5, T6, T7**), testing of the level of defensive reaction after reminder session: **Protocol 3**. Group 3. “Reminder + AN” and group 4 “Reminder + SS”: 9–12 days (**Rest**), rest; 13 day (**Reminder**), reminder session and subsequent injection of anisomycin (AN) or saline solution (SS); 14–18 days (**T3, T4, T5, T6, T7**), testing of the level of defensive reaction after reminder session: **Protocol 3**. Group 3. “Reminder + AN” and group 4 “Reminder + SS”: 9–12 days (**Rest**), rest; 13 day (**Reminder**), reminder session and subsequent injection of anisomycin (AN) or saline solution (SS); 14–18 days (**T3, T4, T5, T6, T7**), testing of the level of defensive reaction after reminder session: **Protocol 4**. Group 5. “AN without reminder” and group 6 “SS without reminder”: 13 day, injection of anisomycin (AN) or saline (SS); 14 day (**T3**), testing of the level of defensive reaction after reminder session.

Testing of the initial level of defensive reaction before the start of elaboration of the conditioned situation reflex in context paradigm (T1) was performed. 1 day after elaboration of conditioned situation context reflex the snails were re-tested (T2) to confirm their learning (Figure 1). Context learning was considered to be elaborated if the response on tactile stimulation of animals in the learning context (on the ball) reliably increased in compare with the defensive reactions under initial testing. Then the testing of the level of defensive reaction after reminder session was performed (T3, T4, T5, T6, T7) (Figure 1).

### Experimental groups

The conditioned reflex to the situation was developed in all animals (*n* = 53), according to the protocol, described in the previous section. Then the animals were separated into 4 groups (Figure 1). In the first group (scheme 1) the animals were injected by p-CPA, then on forth day they received the injection of AN after reminder of the contextual situation (*n* = 12). In the second group (scheme 2) on the fourth day after injection of p-CPA the reminder of the context was made without injection of AN (*n* = 12). In the third group (scheme 3) the reminder of the context and following injection of AN was made without previous injection of p-CPA (*n* = 8). In the fourth group (scheme 3) reminder of the context was made with following injection of SS (*n* = 8). In the fifth group the injection of AN (*n* = 7) were made without the reminder. In the sixth group the injection of SS (*n* = 6) was made without reminder. The reminder of context was the replacement of animals in training context for 20 min, in this case on the ball. The injections by p-CPA were performed 4 days before reminding session, to study the role of 5-HT in reconsolidation and its disruption. Since the maximum depletion of 5-HT by p-CPA was observed after 3–6 days (Popova et al., 1978; Pappius et al., 1988) the time interval for the injection of p-CPA was chosen as 4 days before the reminder. Animals of the first group after a session of “reminder” in contrast to the animals of the second group received injection of p-CPA without AN. Animals of the third and fourth groups were received a session of reminder on the 5 days after learning and then were injected by AN (third group) or SS (fourth group). The testing of animals of first forth groups was done in the next 5 days after reminder and injection (T3–T7). The fifth and sixth groups of snails injected by AN or SS without the reminder were the additional control groups for third and forth groups.

### Data analyses

The results are shown as mean ± SEM. The unpaired Student's *t*-test and non-parametric Mann–Whitney test were used for comparison between two groups. One-way ANOVA followed by Tukey *post-hoc* test and a repeated two-way ANOVA were used for comparison between three- or more statistical groups. Independent *t*-tests and Tukey *post-hoc* test were used to make specific group comparisons. It was used statistical software SigmaStat32. The statistical significance criterion was *p* < 0.05.

## Results and discussion

**Figure 2:**
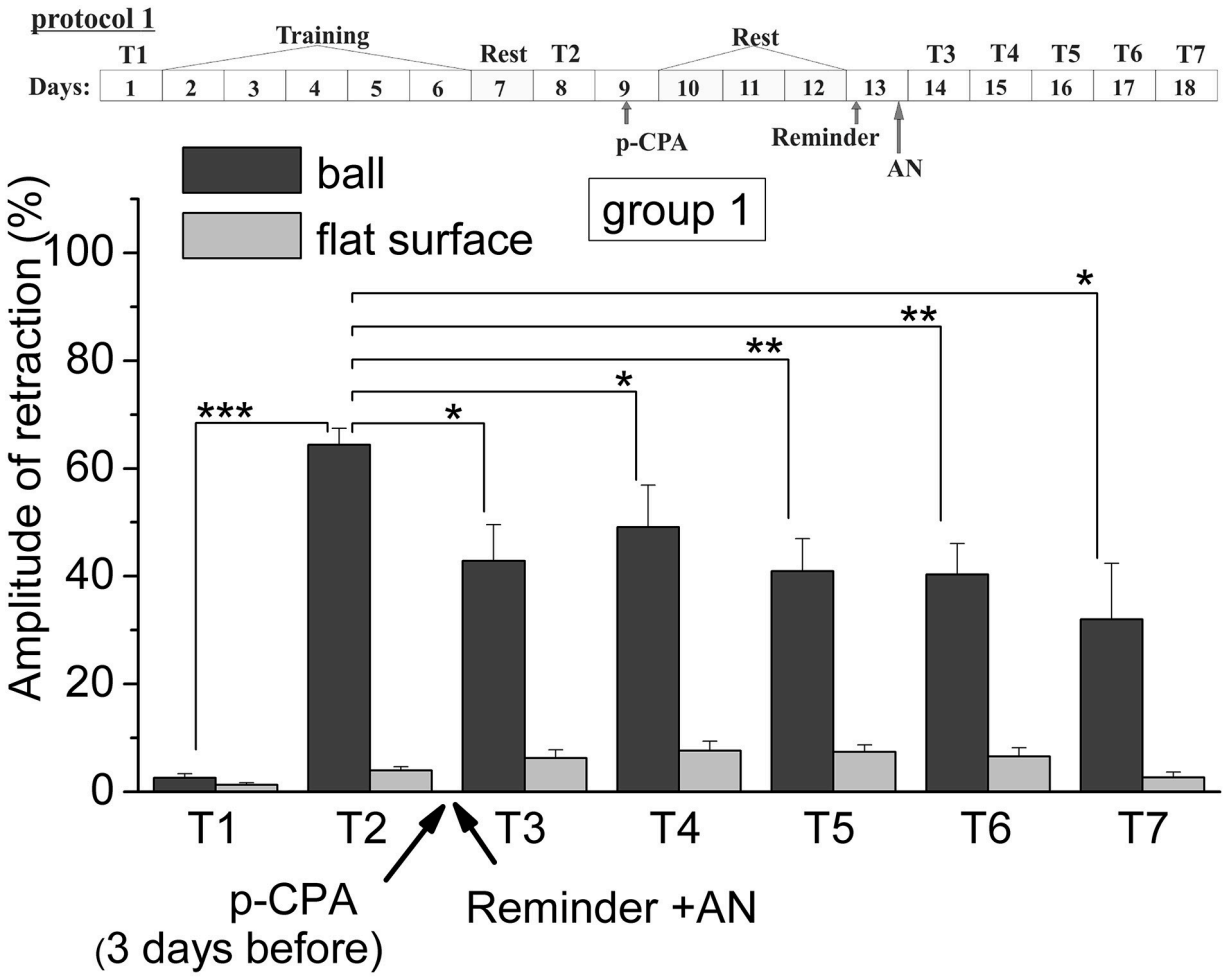
The level of defensive response (the amplitude of response of ommatophores withdrawal) of snails in two contexts, on the ball and flat surface for the first group (“p-CPA + reminder + AN”): an experiment according of protocol no 1: The protocol of experiment is shown in the upper left corner: **T1**, initial testing before the beginning of training; **T2**, testing 2 days after elaboration of conditioned reflex (learning); **T3–T7**, testing of animals after the injections of p-CPA, AN, and reminder on the 3rd−7th day after elaboration of CR (learning): Arrows indicate: **p-CPA**, time of injection of p-CPA (3 day before reminder and injection of AN); **Reminder**, time of Reminder; **AN**, time of injection of AN: Asterisks indicate significant difference of the amplitude of response of ommatophores withdrawal in responses to T3–T7 vs. amplitude of response of ommatophores withdrawal in responses to T2 by paired *t*-test (^*^) equal to *p* < 0.05; (^**^) equal to *p* < 0.01 and T2 vs. amplitude of response of ommatophores withdrawal in responses to T1 by paired *t*-test (^***^) equal to *p* < 0.001: Vertical axis shows value of defensive reaction in response to conditioned stimulus (the amplitude of reaction of ommatophores withdrawal), in % to maximum: Horizontal axis shows the course (protocol) of the experiment: T1, T2, T3–T7, p-CPA, AN, Reminder.

**Figure 3:**
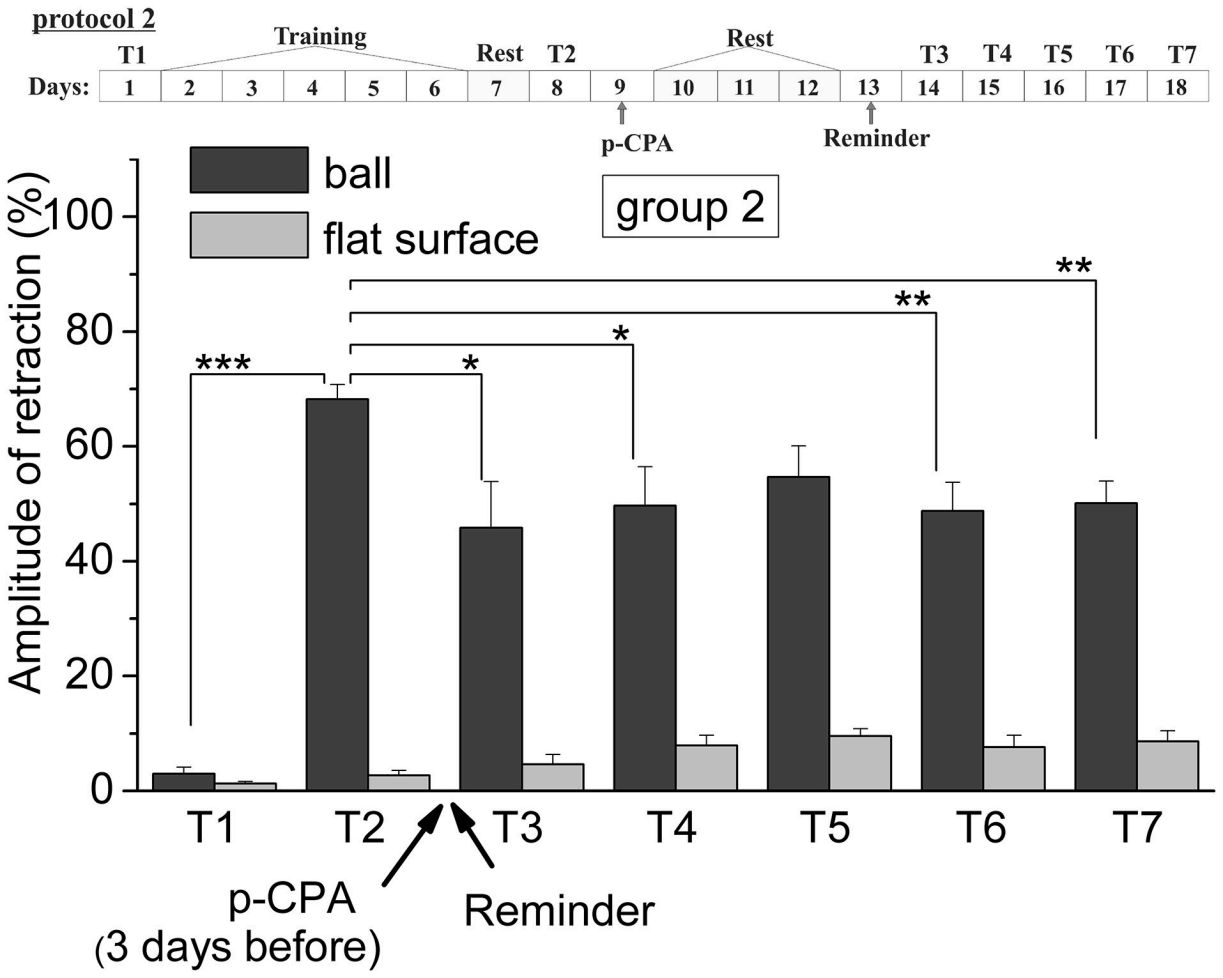
The level of defensive response (the amplitude of response of ommatophores withdrawal) of snails in two contexts, on the ball and flat surface for the second group (“p-CPA + reminder”): an experiment according of protocol no 2: The protocol of experiment is shown in the upper left corner: **T1**, initial testing before the beginning of training; **T2**, testing 2 days after elaboration of conditioned reflex (learning); **T3–T7**, testing of animals after the injection of p-CPA and reminder on the 3rd−7th day after elaboration of CR (learning): **Arrows indicate: p-CPA**, time of injection of p-CPA (3 day before reminder and injection of AN); **Reminder**, time of Reminder: Asterisks indicate significant difference of the amplitude of response of ommatophores withdrawal in responses to T3–T7 vs. amplitude of response of ommatophores withdrawal in responses to T2 by paired *t*-test (^*^) equal to *p* < 0.05; (^**^) equal to *p* < 0.01 and T2 vs. amplitude of response of ommatophores withdrawal in responses to T1 by paired *t*-test (^***^) equal to *p* < 0.001: Vertical axis shows value of defensive reaction in response to conditioned stimulus (the amplitude of reaction of ommatophores withdrawal), in % to maximum: Horizontal axis shows the course (protocol) of the experiment: T1, T2, T3-T7, p-CPA, AN, Reminder.

**Figure 4:**
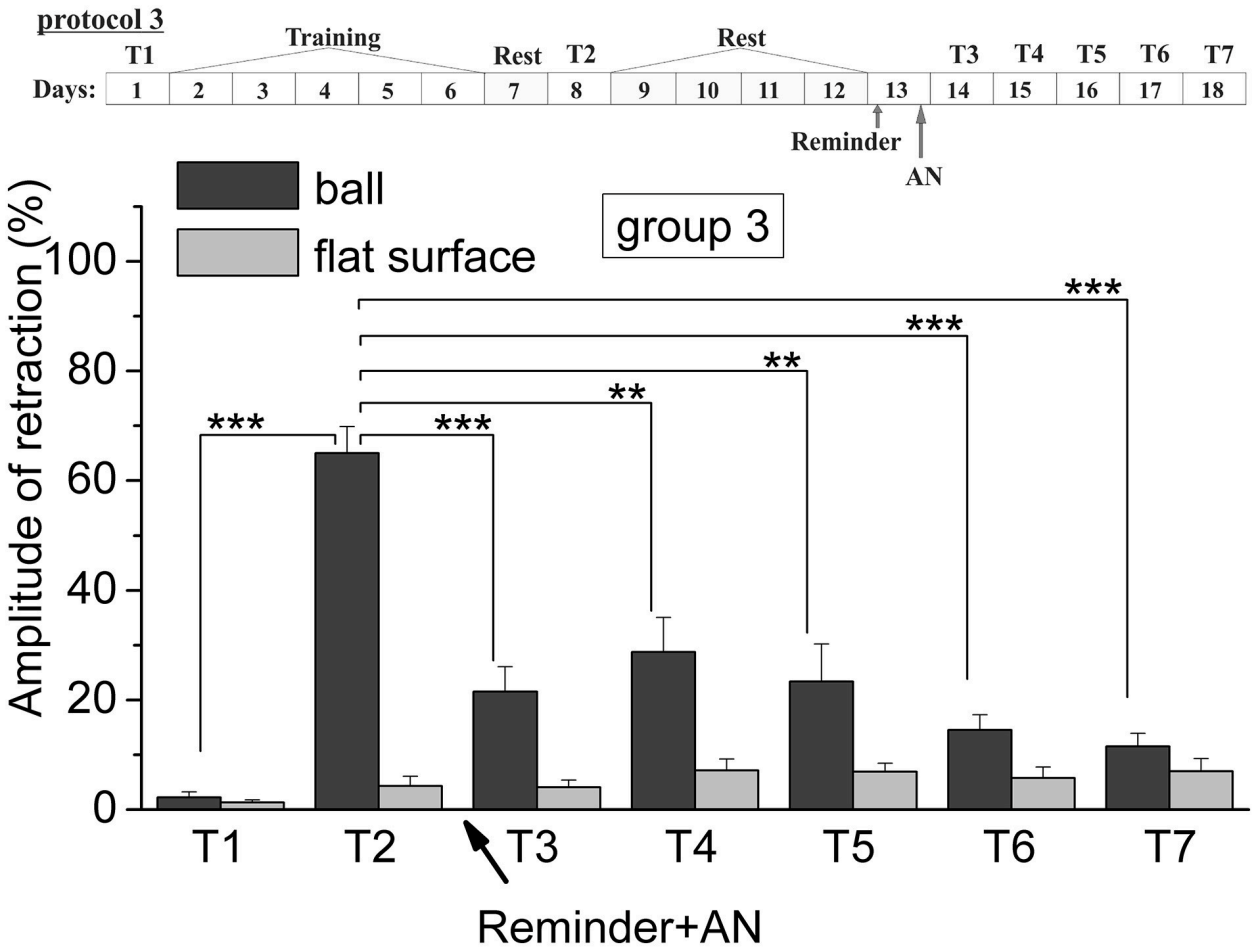
The level of defensive response (the amplitude of response of ommatophores withdrawal) of snails in two contexts, on the ball and flat surface for the third group (“Reminder + AN”): an experiment according of protocol no 3: The protocol of experiment is shown in the upper left corner: **T1**, initial testing before the beginning of training; **T2**, testing 2 days after elaboration of conditioned reflex (learning); **T3–T7**, testing of animals after the injection of AN and reminder on the 3rd−7th day after elaboration of CR (learning); **Arrows indicate: Reminder**, time of Reminder; **AN**, time of injection of AN: Asterisks indicate significant difference of the amplitude of response of ommatophores withdrawal in responses to T3–T7 vs. amplitude of response of ommatophores withdrawal in responses to T2 by paired *t*-test (^**^) equal to *p* < 0.01; (^***^) equal to *p* < 0.001 and T2 vs. amplitude of response of ommatophores withdrawal in responses to T1 by paired *t*-test (^***^) equal to *p* < 0.001: Vertical axis shows value of defensive reaction in response to conditioned stimulus (the amplitude of reaction of ommatophores withdrawal), in % to maximum: Horizontal axis shows the course (protocol) of the experiment: T1, T2, T3-T7, p-CPA, AN, Reminder.

**Figure 5:**
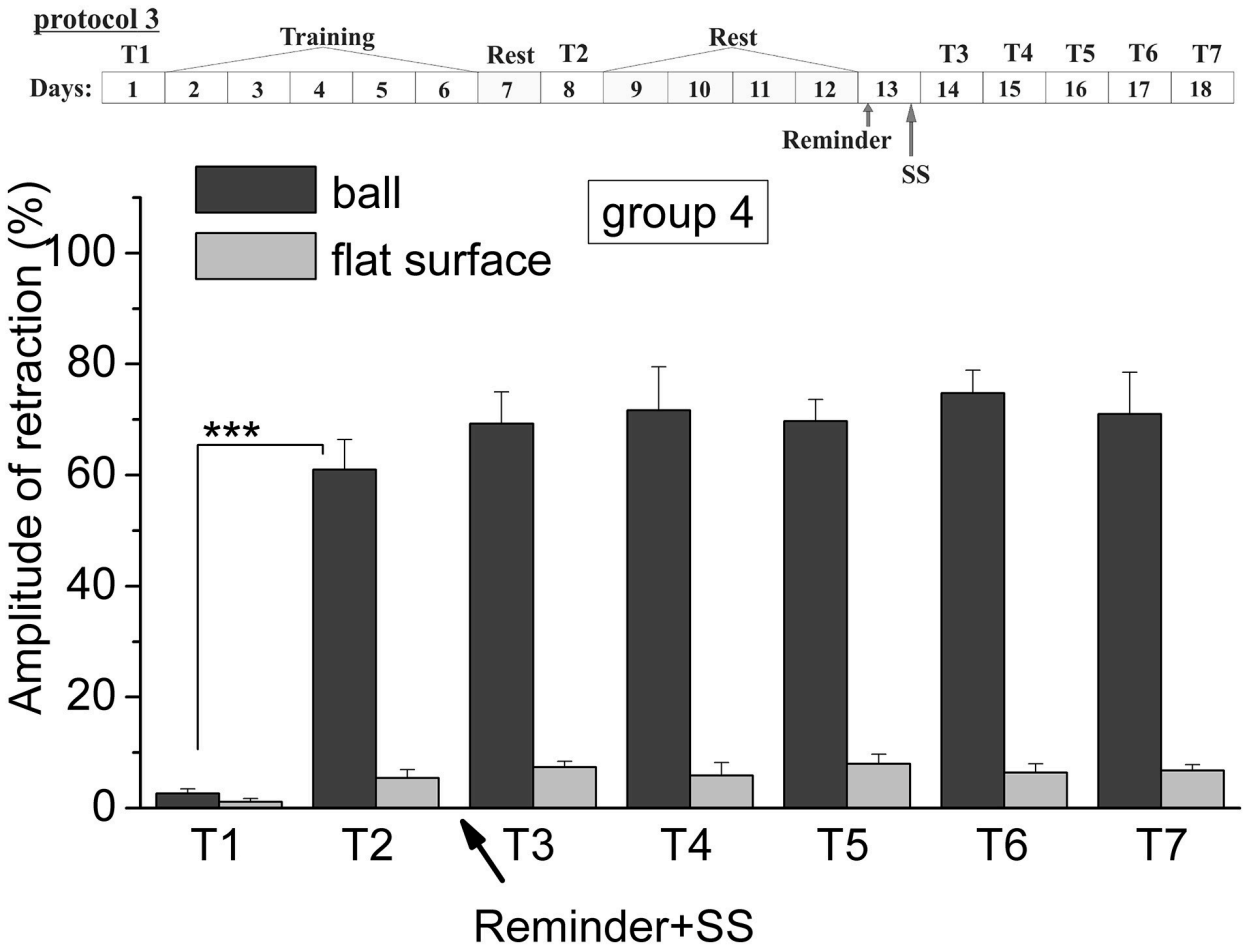
The level of defensive response (the amplitude of response of ommatophores withdrawal) of snails in two contexts, on the ball and flat surface for the forth group (“Reminder + AN”): an experiment according of protocol no 3. “Reminder + SS.” The protocol of experiment is shown in the upper left corner: **T1**, initial testing before the beginning of training; **T2**, testing 2 days after elaboration of conditioned reflex (learning); **T3–T7**, testing of animals after the injection of SS and reminder on the 3rd−7th day after elaboration of CR (learning): Arrows indicate: **Reminder**, time of Reminder; **SS**, time of injection of SS: Asterisks indicate significant difference of the amplitude of response of ommatophores withdrawal in responses to T2 vs. amplitude of response of ommatophores withdrawal in responses to T1 by paired *t*-test (^***^) equal to *p* < 0.001: Vertical axis shows value of defensive reaction in response to conditioned stimulus (the amplitude of reaction of ommatophores withdrawal), in % to maximum: Horizontal axis shows the course (protocol) of the experiment: T1, T2, T3-T7, p-CPA, AN, Reminder.

**Figure 6:**
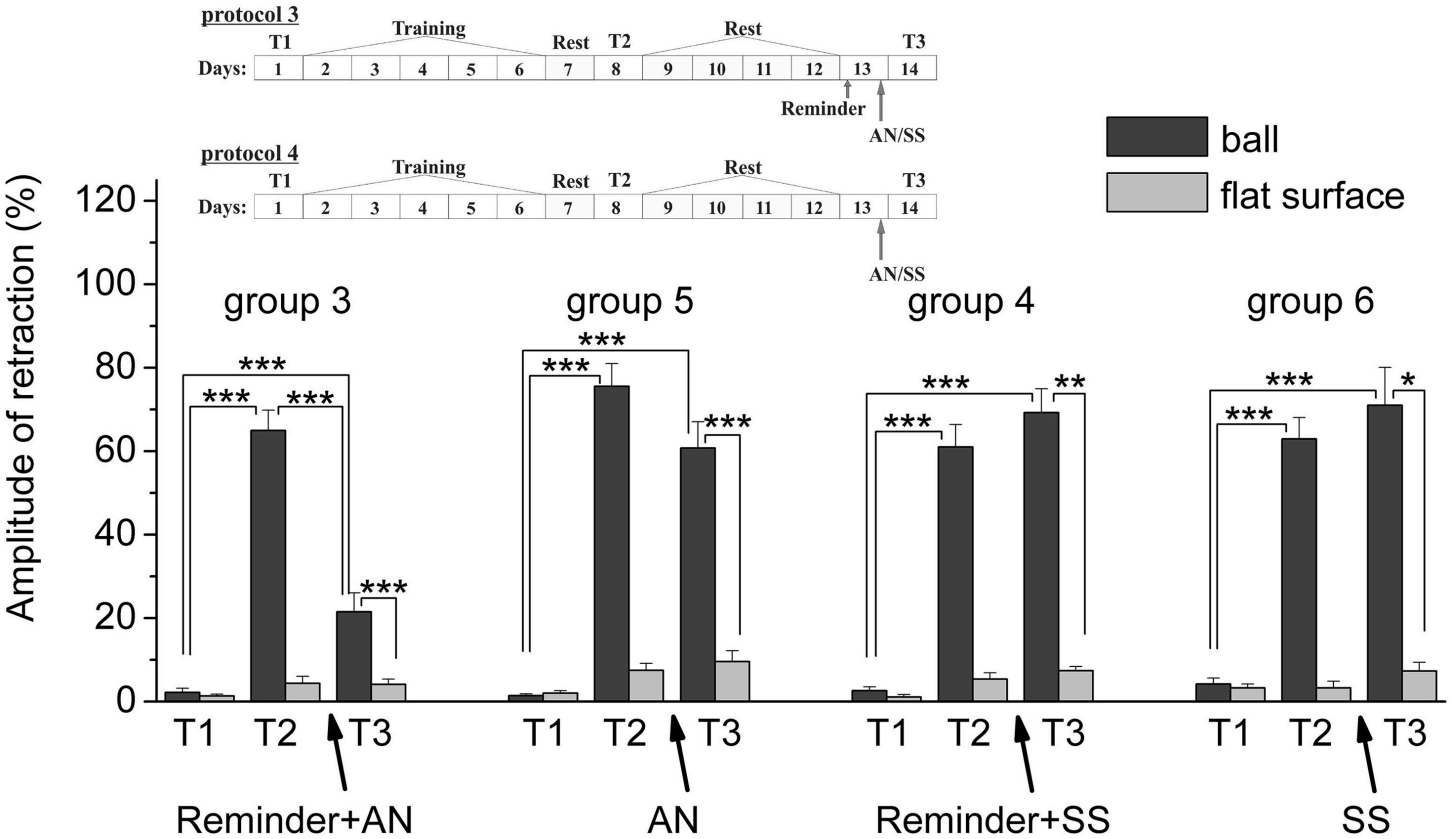
The level of defensive response (the amplitude of response of withdrawal of ommatophore) of snails in two contexts, on the ball and flat surface for the animals of the 5th and 6th groups in comparison with control groups of the 3rd and 4th respectively: an experiments according to protocols no 3 and no 4: The protocol of experiment is shown in the upper left corner: **T1**, initial testing before the beginning of training; **T2**, testing 2 days after elaboration of conditioned reflex (learning); **T3**, testing of animals after the injection of AN or SS on the 3rd day after elaboration of CR (learning): **Arrows indicate: Reminder**, time of Reminder; **SS**, time of injection of SS: Asterisks indicate significant difference of the amplitude of response of ommatophores withdrawal in responses to T2 and T3 vs. amplitude of response of ommatophores withdrawal in responses to T1 by paired *t*-test (^***^) equal to *p* < 0.001; responses to T3 vs. response to T2 by paired *t*-test (^***^) equal to *p* < 0.001; responses to T3 (on flat surface) vs. response to T3 (on ball) by paired *t*-test (^*^) equal to *p* < 0.05; (^**^) equal to *p* < 0.01; (^***^) equal to *p* < 0.001: Vertical axis shows value of defensive reaction in response to conditioned stimulus (the amplitude of reaction of ommatophores withdrawal), in % to maximum: Horizontal axis shows the course (protocol) of the experiment: T1, T2, T3 AN, Reminder, without reminder.

The analysis of results of testing showed that animals of all six groups were successfully learned. The comparing of results of tests from T2 with T1 supported this conclusion. So, the testing (T2) of the levels of defensive reactions of ommatophores retracting in response to tactile stimulation of the anterior part of the foot in animals of 4th group indicated a significant increase in defensive reactions (from 2.6% of the control snails, injected by SS to 61% in experimental group injected by SS) after a training session in context when a snail was on the ball (*p* < 0.001). The statistical analysis of the responses of the 1st group of animals (ball-ball) showed a significant difference between the values of responses before learning T1 (2.6 ± 0.7) and after learning T2 (64.4 ± 3.0) (*P* < 0.001; *t* = 21.155; *n* = 12) (Figure 2). The statistical analysis of the responses of the 2nd group of animals (ball-ball) showed a significant difference between the values of responses before learning T1 (3.0 ± 1.1) and after learning T2 (68.2 ± 2.5) (*P* < 0.001; *t* = 21,312; *n* = 12) (Figure 3). The statistical analysis of the responses of the 3rd group of animals (ball-ball) showed a significant difference between the values of responses before learning of T1 (2.2 ± 1.0) and after learning T2 (65.0 ± 4.9) (*P* < 0.001; *t* = 13,923; *n* = 8) (Figure 4). The statistical analysis of the responses of the 4th group of animals (ball-ball) showed a significant difference between the values of responses before learning T1 (2.6 ± 0.9) and after learning T2 (61.0 ± 5.4) (*P* = 0.001; *t* = 7,763; *n* = 8) (Figure 5). The statistical analysis of the responses of the 5th group of animals (ball-ball) showed a significant difference between the values of responses before learning T1 (1.4 ± 0.4) and after learning T2 (75.6 ± 5.4) (*P* = 0.001; *t* = 13,521; *n* = 7) (Figure 6). The statistical analysis of the responses of the 6th group of animals (ball-ball) showed a significant difference between the values of responses before learning T1 (4.2 ± 1.4) and after learning T2 (61.0 ± 5.1) (*P* < 0.001; *t* = 10,065; *n* = 6) (Figure 6). That is, for all six groups, the significance level of learning reached 0.1%.

Comparison of the levels of defensive reactions of ommatophores retraction in response to tactile stimulation in the situation “on the ball” and on the flat surface provides proof of contextual memory (Gainutdinova et al., 2005; Balaban et al., 2014). It was shown that the training results of the snails of the 4th (injection of SS after the reminder) remained at least 1 week (Figure 5). The statistical analysis of the responses of the 4th group of animals (ball-ball) showed a significant difference between the values of responses before learning T1 (2.6 ± 0.9) and after learning T2 (61.0 ± 5.4) (*P* = 0.001; *t* = 7,763; *n* = 8) (Figure 5). The testing of the same snails on a flat surface had also shown an increase in defensive reactions (from 1.1 ± 0.6 to 5.4 ± 1.4%). The value of defensive reactions in context “on the ball” significantly differed from the value of defensive reactions in context “on flat surface” T7 (71.0 ± 7.5)–T7 (6.8 ± 1.0) (Figure 5) (ball-flat surface) (*P* = 0.001; *t* = 8,105; *n* = 8).

**Figure 7:**
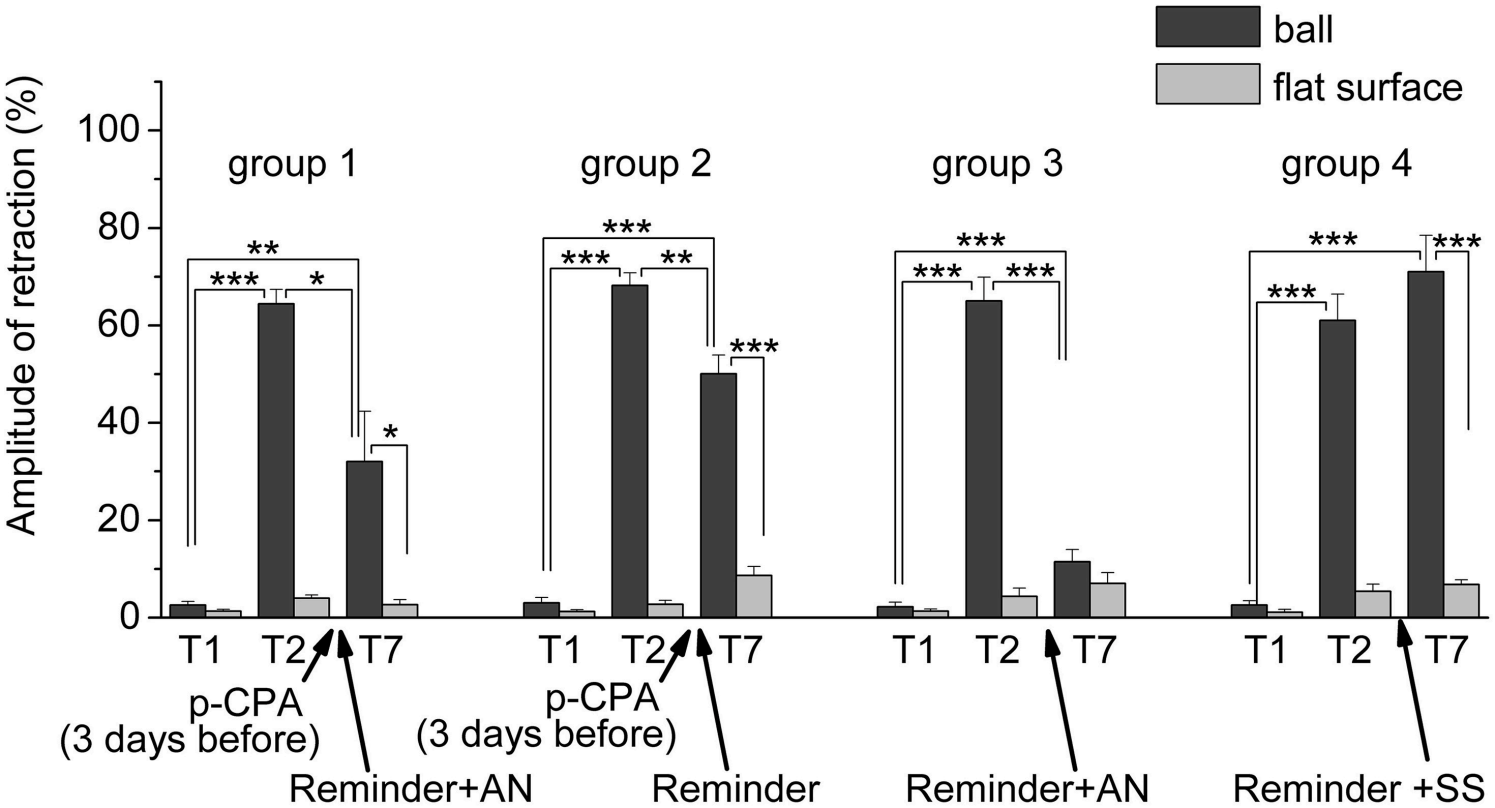
The level of defensive response (the amplitude of response of ommatophores withdrawal) of snails in two contexts, on the ball and flat surface for the all four groups: **A series of experiments:** (1) Protocol 1. Group 1. “p-CPA + reminder + AN”, (2) Protocol 2. Group 2. “p-CPA + reminder”, (3) Protocol 3. Group 3. “Reminder + AN”, (4) Protocol 3. Group 4. “Reminder + SS”: **T1**, initial testing before the beginning of training; **T2**, testing 2 days after elaboration of conditioned reflex (learning); **T7**, testing of animals after the injections of p-CPA, AN, and reminder on the 7th day after elaboration of CR (learning): **Arrows indicate: p-CPA**, time of injection of p-CPA (3 day before reminder and injection of AN); **Reminder**, time of Reminder; **AN** – time of injection of AN; **SS**, time of injection of SS: Asterisks indicate significant difference of the amplitude of response of ommatophores withdrawal in responses to T2 and T3 vs. amplitude of response of ommatophores withdrawal in responses to T1 by paired *t*-test (^**^) equal to *p* < 0.01; (^***^) equal to *p* < 0.001; responses to T3 vs. response to T2 by paired *t*-test (^*^) equal to *p* < 0.05; (^**^) equal to *p* < 0.01; (^***^) equal to *p* < 0.001; responses to T3 (on flat surface) vs. response to T3 (on ball) by paired *t*-test (^*^) equal to *p* < 0.05; (^***^) equal to *p* < 0.001: Vertical axis shows value of defensive reaction in response to conditioned stimulus (the amplitude of reaction of ommatophores withdrawal), in % to maximum: Horizontal axis shows the course (protocol) of the experiment: T1, T2, T3-T7, p-CPA, AN, Reminder.

The repeated testing (T3–T7) of animals of the 1st group showed (Figures 2, 7) that the reminding which was accompanied by the inhibition of protein synthesis and long depletion of brain 5-HT-depo by p-CPA, resulted in a decrease in the level of defensive reactions of snail on the ball. An average value of positive defensive reactions was T2 = (64.4 ± 3.0)%, T3 = (42.9 ± 6.7)%, and T7 = (32.0 ± 10.4)%. The statistical analysis of these group showed a significant difference between the values of reactions before training T1 and after all procedures T7 (*P* = 0.011; *t* = 3,276; *n* = 12). The same memory changing about the context that posed a threat, occurred in the 2nd group of animals (Figures 3, 7), received a reminding on the background of the impaired work of the 5-HT-system, but without inhibition of protein synthesis. The value of the positive defensive reactions on the ball retained on an average were T2 = (68.2 ± 2.5)%, T3 = (45.8 ± 8.1)%, and T7 = (50.1 ± 3.8)%; the statistical analysis showed a significant difference between the values of reactions before training T1 and after all procedures T7 (*P* < 0.001; *t* = 13,301; *n* = 12). However, the level of maintenance of the contextual memory of animals were different. In the 1st group, the statistical analysis showed that the average values were T7 (32.0 ± 10.4) on the ball and T7 (2.6 ± 1.0) on the flat surface (*P* = 0.012; *t* = 3,261; *n* = 12), while in the 2nd group the average values were T7 (50.1 ± 3.8) on the ball and T7 (8.6 ± 1.8) on the surface (*P* < 0.001; *t* = 13,283; *n* = 12).

The testing of animals of the 3rd group 1 day after a reminding of training context and by the subsequent injection of AN showed the significant forgetting of situational conditioned reflex (contextual memory), which continued in following 1 week (Figures 4, 7). The average value were T2 = (65.0 ± 4.9)%, T3 = (21.6 ± 4.5)%, and T7 = (11.5 ± 2.4)%, it was a significant difference between the values before training T1 and after all procedures T7 (ball-ball) (*P* = 0.008; Wilcoxon Signed Rank Test, *n* = 8). The contextual memory of the animals of this group was not remained. There was not significant difference between the value of T7 (11.5 ± 2.4) on the ball and T7 (7.0 ± 2.3) on the flat surface (*P* = 0.281; *t* = 1.167; *n* = 8). The testing of animals of the 4th group, which received the injection of snail saline after a reminder session (the significant differences between T1 and T7 (ball-ball); *P* < 0.001; *t* = 10,289; *n* = 8), demonstrated the persistence of context memory (Figures 5, 7). There was a significant difference between T7 (71.0 ± 7.5) on the ball and T7 (6.8 ± 1.0) on the flat surface (*P* = 0.001; *t* = 8,105; *n* = 8). The testing of these snails on a flat surface on 3-rd day showed an increase in defensive reactions in 1,37 times (T2 (5.4 ± 1.5 and T3 (7.4 ± 1.0) (plane-plane) *t* = 4,340; *P* = 0.003; *n* = 8). The testing of animals of 5th [T2 (75.6 ± 5.4) and T3 (60.8 ± 6.3) (ball-ball) *P* < 0.001; *t* = 9.726; *n* = 7] and 6th [T2 (63.0 ± 5.1) and T3 (71.0 ± 9.1) (ball-ball) *P* < 0.001; *t* = 7.384; *n* = 6] groups 1 day after AN or SS injection without reminder of context has demonstrated the persistence of contextual memory (Figures 6, 7). These experiments demonstrated that the reminder of training situation was a key point of the reconsolidation of context memory. Comparison of the results obtained for 3rd and 5th groups (injection of AN with reminder and without reminder) shows a significant difference in the level of contextual memory in these groups Group3 (T3 = 21.6 ± 4.5)-Group5 (T3 = 60.8 ± 6.3) = ^***^ *t* = 6,333; *P* < 0.001; *n* = 7).

Thus, it was found that the reminding after disrupting of 5-HT system lead to the weakening of contextual memory but not to its forgetting in difference with the third group. Such changing of contextual memory in the case of simultaneous inhibition of protein synthesis and the disrupting of 5-HT system also occur. This effect was significantly different from the direct action of anisomycin, which completely blocked the reconsolidation of contextual memory. The obtained results may indicate that p-CPA used to disruption of the 5-HT system may partially block the signal of “reminder” needed to start the process of reconsolidation. Perhaps for this reason, the disturbance of protein synthesis in the “reminder” in case the first group did not cause a complete blockade of reconsolidation of contextual memory on a situational reflex. We concluded that the serotonin system was included to the process of memory reconsolidation (in our system of situational memory).

The depression and anxiety are complex and heterogeneous disorders of the brain functions (Albert et al., 2014). They greatly change the mentality and affect on the brain functions, one of the manifestations of which are the learning and long-term memory. There is a number of evidence that associates the depression with a decreased activity of the serotoninergic system and support the hypothesis that alterations in serotonin (5-HT) neurons play a role in the pathophysiology of depression (Owens and Nemeroff, 1994; Millan, 2004). However some biochemical theories that link with low levels of 5-HT with depression are no longer tenable. But there are experimental and computational accounts of 5-HT influences on emotional processing throw an intriguing light on the neuropsychology of depression. Therefore, the certain clinical therapy in the treatment of depression is based on the change in the level of serotonin in the body (Byerley et al., 1987; Fickbohm et al., 2005; Winters et al., 2009; Cowen and Browning, 2015).

We have described the data that confirm the existence of a contextual memory in mollusks, which has been shown by us previously (Gainutdinova et al., 2004; Balaban et al., 2014) and other researchers (Child et al., 2003; Kemenes et al., 2006; Lukowiak et al., 2007; Solntseva et al., 2007; Cai et al., 2012; Nikitin and Solntseva, 2012; Dodd and Lukowiak, 2015; Balaban et al., 2016). The experimental results showed that memory reactivation (after reminder) 2 days after learning in our previous studies (Gainutdinova et al., 2005; Balaban et al., 2014) and 5 days after learning in present was a process that sensitive to inhibition of protein synthesis, included in either memory storage or its expression. When long-term memory is reactivated, new protein synthesis requires for its stabilization, as after the initial training (Anokhin et al., 2002; Alberini, 2005; Nader and Hardt, 2009).

The addressing to memory (its expression) is a dynamic process that affects memory by its strengthening, weakening or altering, leading, potentially, to changes in long-term memory (Misanin et al., 1968; Schneider and Sherman, 1968; Lewis, 1979; Przybyslawski and Sara, 1997; Nader et al., 2000; Sara, 2000; Pedreira et al., 2002; Suzuki et al., 2004; Duvarci et al., 2008; Alberini, 2011; Balaban, 2017). In our work it was shown that possibly an important role in retrieval of contextual memory in snail played a modulatory role in 5-HT system. It can be expected that a decrease in the activity of the serotonergic system will affect on the preservation of memory after addressing to it (Nikitin and Solntseva, 2012; Chen et al., 2014; Andrianov et al., 2015; Balaban et al., 2016; Nikitin et al., 2016), for example, in clinical therapy in the treatment of depression based on the change in the level of 5-HT in the body (Byerley et al., 1987). The complex dynamic of the memory reconsolidation process, triggered by the act of reminding, might have important clinical significance. This concerns both and treatment of the specific phobias, and treatment of common emotional disorders, with active involvement in the work of modulatory systems of the brain, as well as during the regulation of protein synthesis in the body.

The authors thank PhD Silantieva D.I. for assistance in design of work.

## Ethics statement

All experimental procedures are in compliance with the Guide for the Car and Use of Laboratory Animals published by the National Institutes of Health, Directive 2010/63/EU of the European Parliament and of the Council of 22 September 2010 and in accordance to guidelines of our Institute. For the experiments the terrestrial snails Helix lucorum were used. The capture of animals in the wild were carried out by competent persons without avoidable pain and distress (Article 9 of Directive 2010/63/EU). Snails transported asleep and then most of them were also stored asleep (Article 33 of Directive 2010/63/EU). Prior to the experiments the snails were kept for no less than 2 weeks in a glass terrarium in a humid atmosphere at room temperature (each group in a separate terrarium) (Article 33 of Directive 2010/63/EU). All groups were housed in separate terrariums which were kept together all the time in the same room under the same conditions.

## Author contributions

ID: behavioral experiments, elaboration of conditioned reflex and reconsolidation of memory about it, statistical processing, participation in determination of tasks for study, in discussion of received results and in writing an article; LM: behavioral experiments, testing animals om all stages of experiment, participation in determination of tasks for study, in discussion of received results; VA: behavioral experiments, injections of drugs, statistical processing, participation in determination of tasks for study, in discussion of received results and in writing an article; KG: determination of tasks for study, statistical processing, participation in in discussion of received results, writing an article.

### Conflict of interest statement

The authors declare that the research was conducted in the absence of any commercial or financial relationships that could be construed as a potential conflict of interest.
